# Harnessing Thermoelectric Power in Self-Healing Wearables: A Review

**DOI:** 10.1021/acsomega.4c10781

**Published:** 2025-02-14

**Authors:** Fatmanur Kocaman Kabil, Ahmet Yavuz Oral

**Affiliations:** † Institute of Nanotechnology, Gebze Technical University, Gebze, Kocaeli 41400, Turkey; ‡ Department of Material Science and Engineering, Gebze Technical University, Gebze, Kocaeli 41400, Turkey

## Abstract

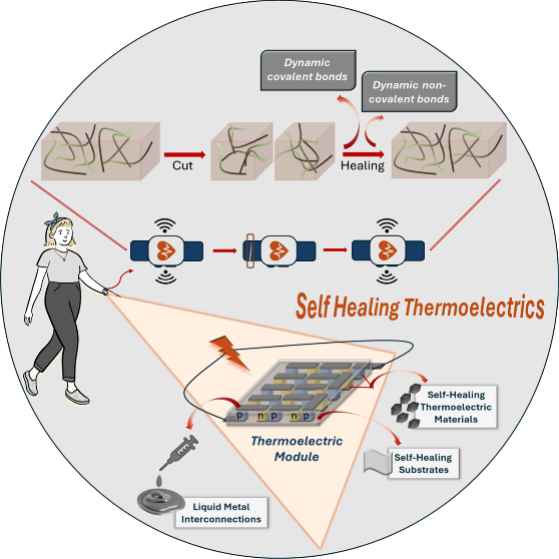

Wearable thermoelectric generators are sustainable devices that generate electricity from body heat to provide a continuous power supply for electronic devices. In healthcare, they are particularly valuable for powering wireless devices that transmit vital health signals, where maintaining an uninterrupted power source is a significant challenge. However, these generators are prone to failure over time or due to mechanical damage caused by mechanical stress or environmental factors, which can lead to the loss of critical healthcare data. To address these issues, the integration of self-healing capabilities alongside flexibility and longevity is essential for their reliable operation. To our knowledge, this review is one of the first to look in depth at self-healing materials specifically designed for wearable thermoelectric generators. It explores the latest innovations and applications in this field highlighting how these materials can improve the reliability and lifetime of such systems.

## 1 Introduction

Especially with the development of Internet-of-Things (IoT) technologies, interest in wearable electronic devices has grown exponentially.^1^ The power required to operate these devices is usually supplied by batteries. However, as batteries are heavy, bulky and require recharging, researchers have turned to more reliable and sustainable power sources.^1^ These wearables provide vital health information such as body temperature, oxygen levels and heart rate.^2^ To ensure complete and continuous monitoring, especially in healthcare systems, uninterrupted and reliable power sources are essential.^3^

The human body offers a promising energy source capable of generating between 100 and 525 W depending on movement, although only a small portion of this energy can be harvested.^4^ Technological advances have made it possible for many devices to operate with power in the mW or even μW range, making the human body an ideal candidate for energy harvesting to achieve a continuous power.^5^ As a result, energy harvesting from the human body and self-powered wearable technologies are becoming a top priority for global research efforts to meet the increasing demand for battery-free health monitoring devices.^1^

Systems such as triboelectric, piezoelectric and thermoelectric generators (TEGs) are used to generate energy.^3^ While piezoelectric and triboelectric generators rely on the movement of the body to generate energy, TEGs use the temperature difference between the body and the surrounding environment and do not require movement. This makes portable TEGs particularly advantageous as they can be seamlessly integrated into electronic devices to provide continuous power without the need for recharging or the threat of a power outage.^6^

The ability of functional materials to heal themselves autonomously is an extraordinary advancement. In recent years, wearable electronic devices have been extensively studied not only for their properties such as flexibility, stretchability and lightweight, but also for their potential for self-healing. If power sources are used for an extended period of time or are mechanically damaged, the reliability of the data transmitted by these devices can deteriorate significantly.^7^ This underlines the critical importance of resilience, especially for devices such as health monitors that need to operate continuously. Self-healing wearable TEGs offer a solution by providing the uninterrupted power needed for such applications.

While metals and ceramics can exhibit self-healing properties, polymers dominate as primary materials for self-repairing technologies. These materials can either fully or partially restore their original performance without the need for manual intervention. This process is activated by localized damage, such as a drop in performance in a specific area of the material. The success of self-healing is measured by the healing efficiency, which indicates the percentage of the material’s original mechanical, thermal or electrical properties that are restored — 100% efficiency means full recovery.^8^

In thermoelectric devices, self-healing mechanisms are crucial due to their influence on critical properties such as figure of merit, power factor, Seebeck coefficient, electrical and thermal conductivity. Furthermore, the combination of self-healing capabilities with mechanical stretchability is essential for the development of advanced long-lasting wearable thermoelectric devices. The development of thermoelectric materials that combine flexibility, stretchability and self-healing would be a transformative step to meet the increasing demand for reliable, wearable, self-powered devices.^9^

This review is important as it is the first report to thoroughly analyze the self-healing materials in wearable TEGs. It concurrently illuminates the current state of the art in self-healing materials used in thermoelectric devices and provides insights into the latest advances and applications so that the reader is aware of the latest research and developments. In addition, it identifies gaps in current knowledge and research and serves as a roadmap for future studies in the rapidly evolving field of wearable technologies and self-healing materials.

## 2 Working Principle of Wearable Thermoelectric Generators

TEGs convert waste heat directly into electricity or vice versa.^10^ These devices work on the basis of the Seebeck effect. The Seebeck effect was discovered by Thomas Johann Seebeck in 1821.^5^ The Seebeck coefficient (often referred to as *S*) is a measure of the magnitude of an induced thermoelectric voltage in response to a temperature difference in a material. The Seebeck coefficient is an intrinsic material property and is of central importance to the operation of TEGs and Peltier devices. Mathematically, the Seebeck coefficient can be defined by the following formula:
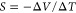
1where *S* is the Seebeck coefficient, *ΔV* is the voltage difference (or Seebeck voltage) generated across the material and *ΔT* is the temperature difference across the material. The negative sign means that the direction of the induced voltage is opposite to the temperature gradient. The unit of the Seebeck coefficient is volts per kelvin (*V/K*). The value of *S* can be positive or negative, depending on which type of charge carrier predominates in the material (positive for holes in p-type materials, negative for electrons in n-type materials). The total thermoelectric voltage generated in a circuit of two different materials (as in a thermocouple) is determined by the difference in the Seebeck coefficients of the two materials:
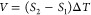
2where *S*_1_ and *S*_2_ are the Seebeck coefficients of the two materials. Materials with a high absolute value of the Seebeck coefficient are generally preferred in TEGs.^11^

The efficiency of thermal energy conversion is determined by figure of merit (ZT) value.^4^ The formula for this dimensionless ZT value is defined in eq 3.^12,13^
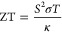
3where *S*, σ, *T* and *κ* are the Seebeck coefficient, electrical conductivity, absolute temperature and thermal conductivity, respectively. To obtain high ZT values, the materials should have high *S*, σ, *T* and low *κ*. Since *S*, σ and *κ* are interrelated, it is challenging to meet these conditions altogether in a thermoelectric material. For example, if the Seebeck coefficient is increased, the electrical conductivity decreases with decreasing carrier concentration.^14^

For polymer-based thermoelectric materials, the power factor (PF) is commonly used as a key performance indicator instead of the ZT, which is more applicable to inorganic thermoelectrics.^15^ This is because ZT depends on both thermal and electrical conductivity and the inherently low thermal conductivity of polymers minimizes its relevance. Due to this property, PF, which focuses on electrical conductivity and Seebeck coefficient, is a more appropriate metric to express the efficiency of polymer-based thermoelectrics.^15^ The formula for the power factor is as follows:
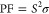
4

**Figure 1:**
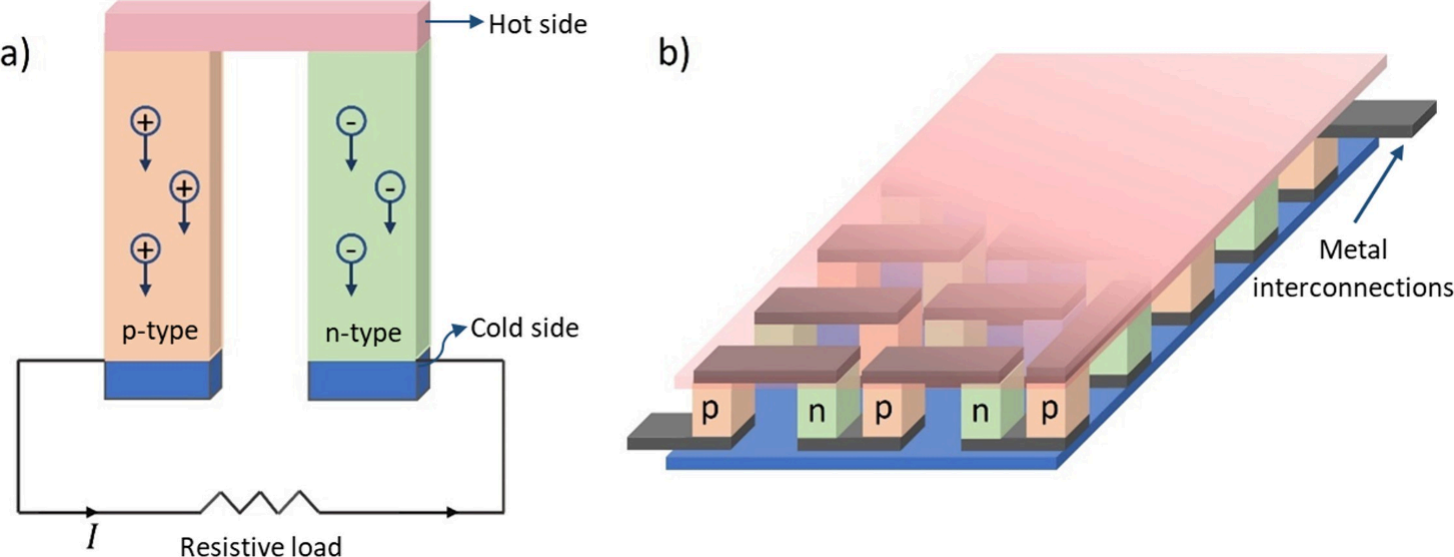
Schematic view of (a) thermoelectric element (b) thermoelectric module.

TEGs consist of p-type materials, which have a majority of positive charge carriers (holes), and n-type materials, which have a majority of negative charge carriers (electrons).^3^ To increase the output power of the generator, multiple p-type and n-type materials are thermally connected in parallel to facilitate heat transfer and electrically connected in series to maximize the output voltage.^5^Figure 1 shows schematic views of basic thermoelectric element and conventional thermoelectric module.

To make the device wearable and adapt it to the curved shape of the human body, flexible TEGs are usually preferred to rigid ones.^5^ Flexible thermoelectric devices often use organic materials such as conductive polymers, carbon-based materials, composites or organic–inorganic hybrids.^16^ However, in recent years, inorganic materials with superior thermoelectric performance have also been integrated into flexible devices by incorporating them to flexible substrates or bonding them with stretchable interconnects.^17−19^

Over time, significant advancements in thermoelectric devices have been achieved through the exploration of innovative materials such as nanostructured, organic, and hybrid compounds, which have greatly improved device performance, scalability, and power output. The integration of advanced manufacturing methods has further expanded the industrial applications of thermoelectric technology. Moreover, the development of novel materials, along with the optimization of existing ones using advanced synthesis and characterization techniques, continues to drive progress in the field. Additionally, new structural designs, such as thinner and more flexible film structures, have further enhanced the efficiency of TEGs.^20^

## 3 Fabrication Processes of Wearable Thermoelectric Generators

Wearable TEGs are typically manufactured in a series of steps. These include the synthesis and fabrication of thermoelectric legs, integrating them with interconnects, fabrication of electrodes, and encapsulation of the device in a flexible polymer for durability and conformability.^21^

**Figure 2:**
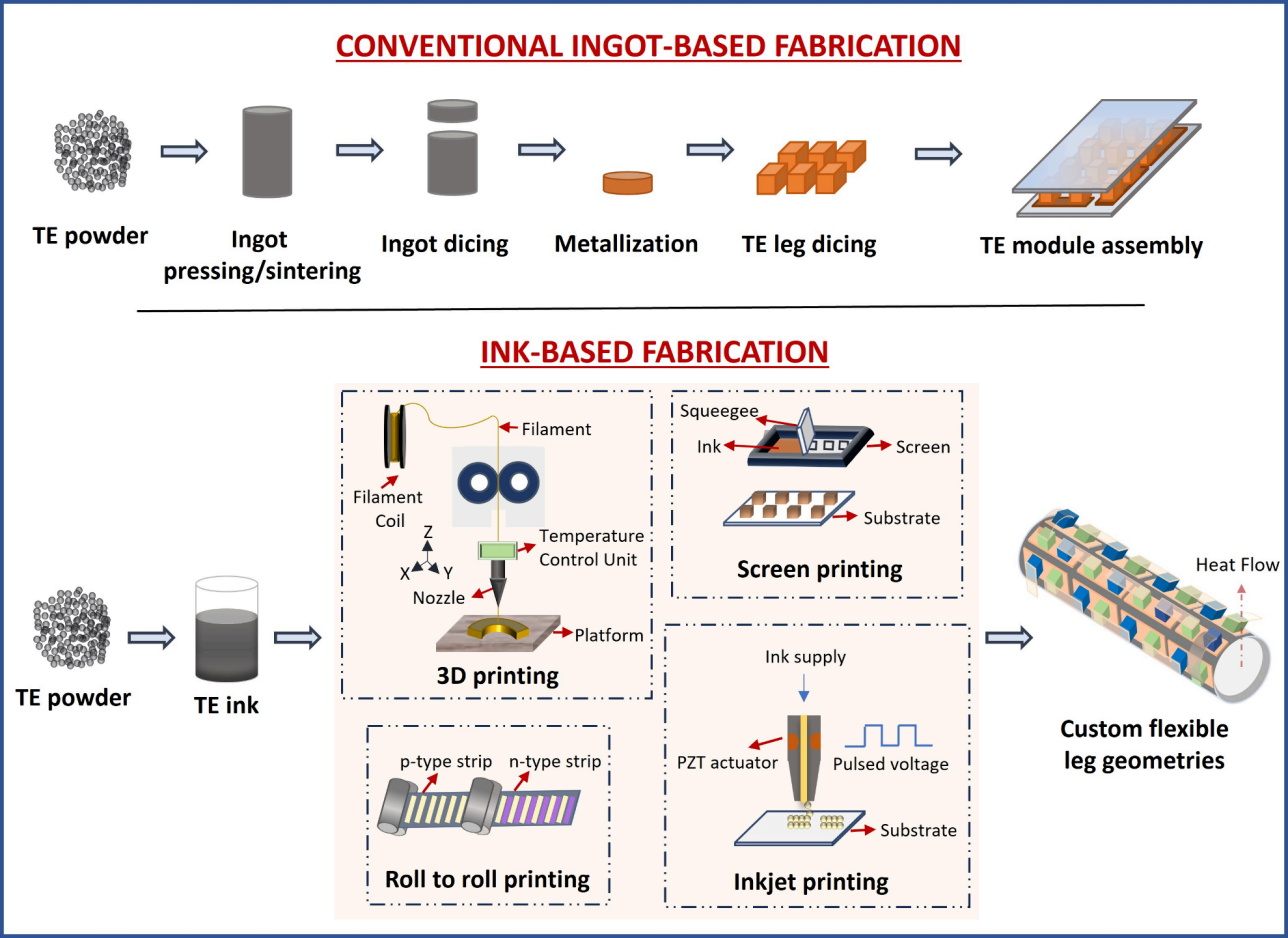
Flowcharts of the ingot and ink-based manufacturing processes of TEG.^22^ Adapted with permission from ref (22). Copyright 2018 John Wiley and Sons.

TEGs can be fabricated by ingot-based or ink-based processes.^22^Figure 2 shows the ingot and ink-based manufacturing processes of TEGs. Ink-based printing technologies, such as 3D printing, roll-to-roll printing, inkjet printing, and screen printing, have been widely used. Additive manufacturing (3D printing) is a bottom-up approach that enables the creation of complex geometries from three-dimensional model data, transforming the production of thermoelectric materials and devices by allowing them to be fabricated on flexible and nonflat surfaces.^23,24^ This advancement improves their adaptability and makes them ideal for applications such as wearable electronics and conformal designs. As a result, TEGs can be seamlessly integrated into wearable devices where flexibility and adaptability to various shapes are essential. Roll-to-roll printing has gained popularity in thermoelectric device manufacturing in recent years due to its compatibility with flexible surfaces, its cost efficiency and its ability to operate without a vacuum environment.^25^ In addition to this, inkjet and screen printing are widely used techniques for fabricating thermoelectric materials, each offering distinct benefits. While inkjet printing enables high-resolution, precise thin films with minimal material waste, making it ideal for fine patterns, screen printing excels in producing thick films on a larger scale, providing a more cost-effective solution despite its limitations in pattern resolution.^26^ These manufacturing processes, particularly in thin-film fabrication, offer significant advantages such as lower material costs, improved thermal gradient retention, compact size, and lightweight construction. These properties make thin-film TEGs compatible with flexible substrates and enable the fabrication of transparent devices. As a result, TEGs are considered promising candidates for a range of applications, particularly in wearable technologies and the IoT.^27^

Although ingot-based processes are costly and involve time-consuming steps such as synthesis, cutting, metal capping, assembly and soldering, they continue to be used due to the superior thermoelectric performance of ingot-based materials compared to ink-based alternatives.^22,28^ Therefore, studies continue to be conducted to develop flexible devices using traditional ingot-based materials with high thermoelectric performance.

## 4 Self-Healing Mechanisms

Minor wounds or injuries to the human skin can often heal spontaneously under normal physiological conditions. Inspired by this biological phenomenon, researchers have explored the applicability of self-healing properties in electronic devices.^7^ Soft electronic materials with self-healing capabilities enable devices to restore critical functions such as electrical conductivity or mechanical integrity after unexpected damage.^28^ This not only improves the durability of the devices, but also significantly extends their service life.^29^ As electronic devices are increasingly integrated into daily life, particularly in wearable and flexible technologies, the need for self-healing capabilities has grown. Self-healing electronic materials are used in a wide range of applications, including energy harvesting and storage devices,^30−32^ sensors,^33−35^ and transistors.^30,36−38^

**Figure 3:**
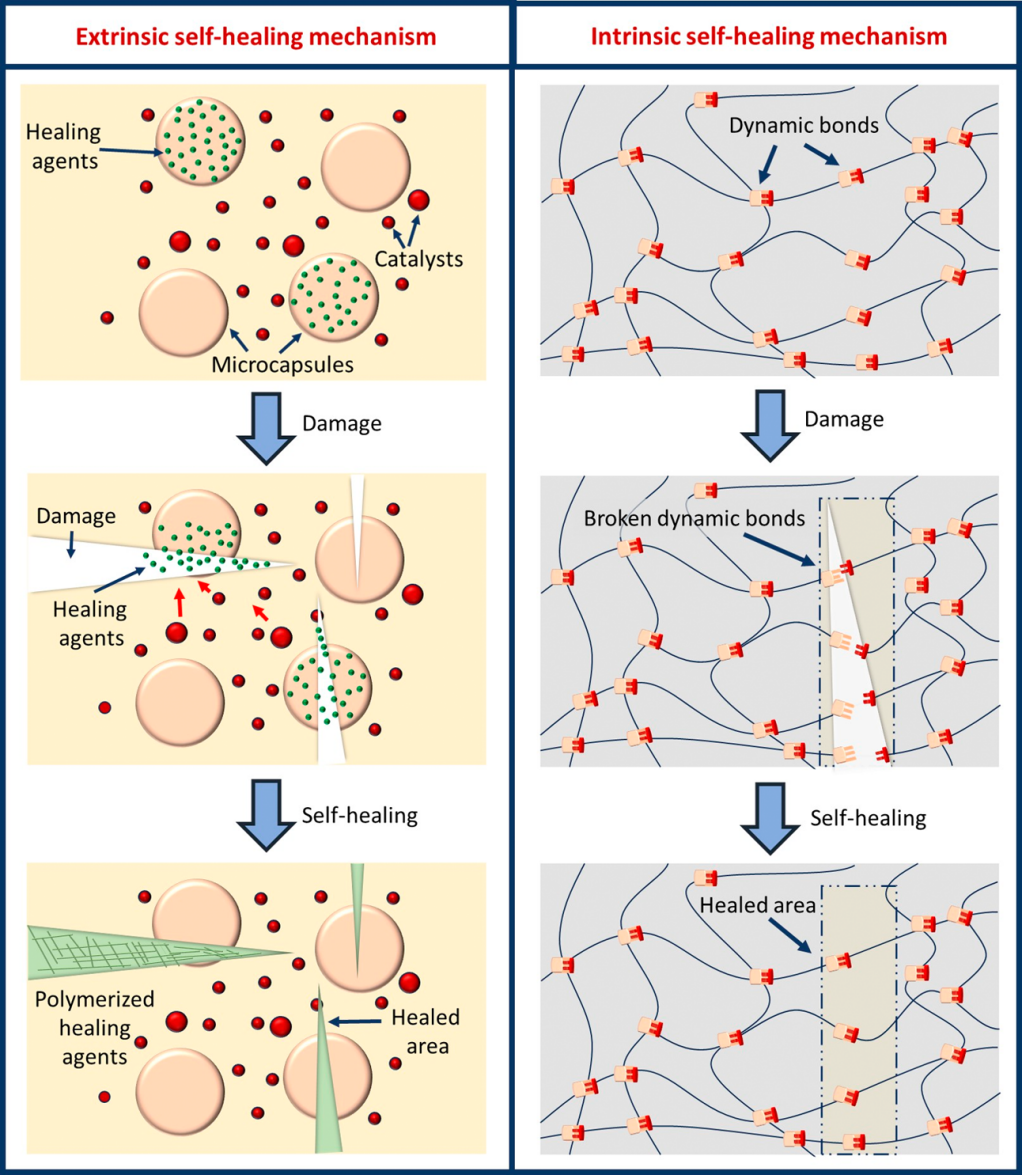
Schematic representations of extrinsic and intrinsic healing mechanisms.^7^ Adapted with permission from ref (7). Copyright 2021 John Wiley and Sons.

Self-healing systems are roughly divided into two groups: automatic and nonautomatic. In automatic systems, healing occurs spontaneously when damage occurs, while in nonautomatic systems an external stimulus, such as heat or light, is required for the healing process.^39^ Additionally, self-healing materials are classified as either intrinsic or extrinsic based on their healing mechanisms. Intrinsic materials possess inherent self-healing properties, while extrinsic systems rely on external healing agents.^40^Figure 3 shows the intrinsic and extrinsic healing mechanisms.^7^

Extrinsic self-healing materials typically utilize healing agents encapsulated in microcapsules, nanocapsules or vascular networks within a polymer matrix.^41^ When damage occurs, these capsules rupture and release the healing agents to initiate a chemical reaction that repairs the material. However, capsule-based systems have their limitations as they can only heal a material a limited number of times and lose their functionality if repeated damage occurs at the same site.^42^ Therefore, vascular self-healing systems have been developed in which healing agents are stored in capillary channels and continuously delivered to the damaged areas through a microvascular network.^39^ Although these systems enable repeated repairs, they face challenges such as complex fabrication and limited repair cycles.

Extrinsic methods offer rapid repair but are hindered by the selection of compatible healing agents, high production costs and potential deterioration of host material properties.^43^ The efficiency of these systems depends on the size and quantity of the curative and its compatibility with the shell material. Smaller containers allow for faster and more localized repairs, while larger containers deliver larger amounts of healing agents. For repeated self-healing, vascular and fiber-based designs are preferred over capsule-based systems for single use. Innovative approaches, such as bimodal distribution of agent sizes, combine electrospray-produced nanocapsules with conventional microcapsules to improve repair efficiency while reducing costs. This approach is promising to improve self-healing capabilities and ensure cost-effective solutions for advanced applications.^44^

Intrinsic self-healing materials rely on molecular interactions and not on external healing agents. They allow multiple repairs without being limited by the availability of healing agents. However, these materials require specific temperatures for effective healing and their production must be tailored to the desired properties and application environment.^39,40^ Healing occurs through the regeneration of dynamic covalent and noncovalent bonds or the diffusion of polymer chains. Dynamic covalent bonds, such as those formed by Diels–Alder reactions, disulfide bonding, and imine bonding, are essential for enabling self-healing properties. Similarly, noncovalent interactions like hydrogen bonding, electrostatic forces, and metal coordination also contribute significantly to the self-healing process.^45^

Dynamic covalent bonds are particularly valued for their ability to form reversible bonds, giving polymeric materials flexibility, self-healing ability, tunable mechanical properties and reprocessing potential.^43^ The rate of self-healing is influenced by the mobility of the polymer chains, the concentration of broken dynamic bonds and the activation energy required for bond exchange.^40^ External stimuli, such as heat, light or catalysts, can increase the mobility of the polymer chains and accelerate the healing process.^30^ These externally triggered systems have a nonautonomous self-healing mechanism.

Intrinsic self-healing polymers generally have a low glass transition temperature, which facilitates the movement of the polymer chains and contributes to the self-healing process. The enhancement of dynamic interactions, such as hydrogen bonding and pi-pi stacking, further increases the self-healing capacity.^40^ For these reasons, intrinsic self-healing materials are preferred over extrinsic ones in soft electronics. They offer greater flexibility and adaptability for advanced applications.

Despite significant advances in self-healing materials, two major challenges persist: the lack of standardized protocols for evaluating and comparing the healing efficacy of different systems and concerns about the scalability and performance of these materials in practice. For real-world use, it is critical to thoroughly evaluate and optimize factors such as fatigue resistance, thermal and electrical conductivity, and overall material stability.^46^

Ongoing efforts are aimed at developing the ultimate self-healing system, with future research focusing on innovative polymeric materials and mechanisms to improve the effectiveness of self-repair.^47^ Researchers are also exploring novel approaches, such as visual self-healing indicators that trigger a color change upon damage, to go beyond traditional mechanisms. To gain a deeper understanding of these processes, a combination of experimental studies, simulations and numerical modeling is required. Such an integrated approach is essential to optimize self-healing in functional polymer coatings and other advanced applications.^48^

## 5 Self-Healing Thermoelectric Generators

Self-healing materials can be electrical conductors (liquid metals etc.), ionic conductors (polymer electrolytes etc.), insulators (polymers etc.), or semiconductors.^30^ In TEGs, self-healing materials are used in three main parts: the part that converts heat into electricity, the substrate and the connections between the parts. This section looks at the different types of self-healing materials used in TEGs.

### 5.1 Liquid Metal Interconnections

Soft electronics is a groundbreaking innovation in electronic devices. It uses durable and flexible materials, many of which are capable of self-healing.^40^ These materials have the ability to repair themselves, even under mechanical stress, so that their functionality is maintained. Liquid metals are one of the self-healing materials that can be used as conductors in soft electronics. These metals melt at temperatures below room temperature and are used in a liquid state.^49^

Bismuth telluride-based inorganic materials with high ZT values are often preferred in TEGs.^1^ However, for the device to be used as a wearable, it must have a flexible structure that is compatible with the curved structure of the human body. Despite the growing popularity of organic materials such as carbon-based substances for flexible applications, it remains a challenge to achieve the thermoelectric conversion efficiency of inorganic materials. Therefore, in recent years, liquid metal materials have been used as a link between inorganic materials in bulk or thin film to connect them and give them flexibility.^50^

Gallium-based liquid metals are preferred over mercury due to their low toxicity and negligible vapor pressure, making them safe for handling. An intriguing characteristic of these metals is their surface oxide layer, which allows them to be molded into useful shapes through techniques like fluidic injection and 3D printing.^51^ A flexible and wearable thermoelectric device that can repair itself and uses eutectic gallium indium (EGaIn) as a liquid metal compound between thermoelectric materials was first developed in 2017.^17^ In the same study, high ZT bismuth-based semiconductor legs (Bi_0.5_Sb_1.5_Te_3_ as p-type, Bi_2_Se_0.3_Te_2.7_ as n-type) were connected with liquid metals to impart both ductility and self-healing properties to the device. Since the device needs to be encapsulated after the liquid metal compounds are fabricated, it was embedded in PDMS, a stretchable elastomer. It was found that three different resistances, namely the electrical resistance of the thermoelectric legs, the resistance of the EGaIn junctions and the contact resistance between them, have an influence on the efficiency of the thermoelectric devices. With a melting point of 15.5 °C, EGaIn shown to increase the efficiency of the device because it has a very low electrical resistance (2.94 μΩ cm). In this study, the device was tested on the human body without the use of a heat sink. Over a long period, it was observed that the device’s temperature decreased as the body absorbed its heat, leading to a reduction in the device’s output power. It is therefore predicted that a device with better performance can be developed if flexible heat sinks that are compatible with the human body are used.^17^

Zhu et al.^50^ created a flexible, recyclable, and self-healing thermoelectric device by utilizing EGaIn liquid metal interconnects to connect inorganic thermoelectric legs (Bi_2_Te_3_ and Sb_2_Te_3_). In this study, thermoelectric materials were coated onto polyimine using a screen-printing technique. In addition to the dynamic covalent thermoset polyimine layer used as an encapsulating layer, the excellent flowability of liquid metal electrodes enabled the flexible thermoelectric device to exhibit remarkable self-healing capabilities in response to electrode disconnection. In cases of minor damage, the device could effortlessly reconnect due to the liquid metal’s excellent flowability. Additionally, the nature of liquid metals eliminated fatigue issues and prevented stress concentration at the interface between the liquid metal and the rigid thermoelectric legs, guaranteeing a robust and reliable interconnection even under severe mechanical deformation. It was observed that the device fully repaired itself within 24 h. Following the healing and recycling processes, the device demonstrated output performance on par with the original devices. Additionally, the device showed notable reliability and stability when subjected to cyclic deformation.

Chen et al.^52^ developed the first flexible, inkjet-printed TEG. In their study, Bi_2_Te_3_ and Bi_0.5_Sb_1.5_Te_3_ nanowires were used as p- and n-type thermoelectric materials. Usually, inorganic materials are used in TEGs in the form of pellets. In this study, these inorganic materials were coated on polyimide using the solution-based inkjet printing technique. They used liquid metal to make the connection between the p- and n-type legs. They showed that TEGs with EGaIn contacts, which provide the connection between the p- and n-type legs of the TEG, are more flexible than devices with silver paste connections. Thanks to solution-based printing technology and liquid metal interconnections, they have developed a thermoelectric thin-film device with high durability and flexibility.

Han et al.^53^ reported 3D printed, highly stretchable and self-healable thermoelectric devices for wearable applications. They used a liquid metal elastomer composite (EGaIn and Ecoflex 00-30) as thermal interface layer, and EGaIn liquid metals as interconnects. The elastomer Ecoflex 00-30 also served as an encapsulation material and combines flexibility with effective thermal management and structural support. Rigid p-type and n-type Bi_2_Te_3_ thermoelectric materials are embedded within a flexible matrix to ensure high energy conversion efficiency along with mechanical flexibility. The device has been designed with various infill ratios and thermal insulation structures to maximize heat retention and conversion efficiency at low temperature gradients, which improves its suitability for body heat applications. The device also can endure up to 230% strain without electrical failure and retains its functionality over 2000 stretching cycles at 50% strain, proving its robustness for wearable electronics. They have also integrated a printed stretchable heat sink to improve thermal management and significantly increase power density in forced air convection tests. Their thermoelectric device has successfully powered LEDs and sensors directly from body heat, demonstrating the potential for self-sufficient wearable applications that do not require batteries.

Liquid metal composites are also being investigated for the development of innovative and durable materials in soft electronics, with a particular focus on their self-healing, reconfigurable and recyclable properties. The composite material, in which liquid metal droplets are embedded in a stretchable, conductive polymer matrix, not only offers self-healing properties and forms conductive networks to improve longevity and recyclability, but also exhibits excellent electromechanical properties. In the future, these composites could lead to more resilient, flexible electronics that repair themselves. This opens up possibilities for long-lasting wearable devices and serves as a potential power source for the next generation of soft robotics.

### 5.2 Flexible Substrates

**Figure 4:**
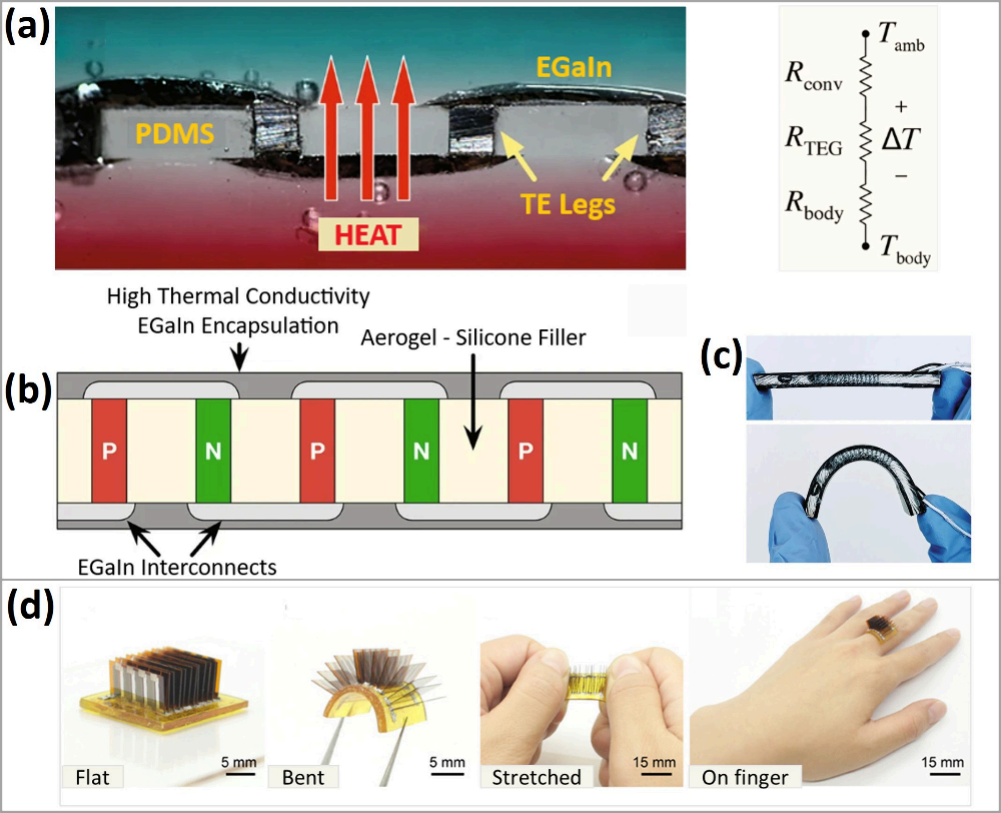
(a) Cross-sectional image of a flexible TEG developed by Ramesh et al.,^18^ showing liquid metal connections for enhanced flexibility. Reprinted from ref (18). Copyright 2021 The Authors. (b) Schematic diagram illustrating the key components of the flexible TEG and their arrangement. (c) Demonstration of the flexible TEG’s adaptability as it is held between two fingers, highlighting its mechanical flexibility. (d) Stretchable end self-healing wearable TEG developed by Ren et al.^56^ Reprinted from ref (56). Copyright 2021 The Authors.

For TEGs, the goal is to have leg materials with low thermal conductivity, while the substrate or encapsulation material that comes into contact with the human body should ideally have high thermal conductivity.^54^ In this regard, a team of researchers created a device featuring a thermoelectric leg and liquid metal compounds. They encased this device in an elastomer made of graphene nanoplatelets, EGaIn and PDMS, which is known for its high thermal conductivity, to improve the device’s efficiency in converting heat.^19^ Zadan et al.^55^ created a durable device that can stretch up to 50% without electrical or mechanical damage. They achieved this by using a thermally conductive elastomer layer containing liquid metal. Another study^18^ aimed to make the temperature difference between the hot and cold sections of the thermoelectric legs larger. To do this, they built a wearable device with EGaIn liquid metal compounds and a heat-conducting cover. They also used an aerogel-PDMS composite, a material with very low heat conductivity, as a filler between the legs (Figure 4a–c).^18^ The resulting TEG with a power density of 35 μWcm^–2^ can be used to power many low-power electronic devices. Table 1 summarizes recent studies on self-healing wearable TEGs.

**Table 1:** Self-Healing TEGs

**Thermoelectric Materials**									
**P-type**	**N-type**	**Healing Mechanism**	**Encapsulation Layer/Substrate**	**Bending/Stretching Cycle**	**Bending Radius (mm)**	**Power Density** (μW/cm^2^)	**Δ***T*	**Performance After Healing**	**Ref**
Bi_0.5_Sb_1.5_Te_3_	Bi_2_Se_0.3_Te_2.7_	Liquid Metal (EGaIn)	PDMS	1000	5	11.57	1.1	No change in resistance	Suarez et al.,^17^ 2017
Bi_0.5_Sb_1.5_Te_3_	Bi_2_Se_0.3_Te_2.7_	Liquid Metal (EGaIn)	Graphene nanoplatelet/EGaIn/PDMS	250	17	5.2	∼10	No change in resistance	Sargolzaeiaval et al.,^19^ 2020
Bi_0.5_Sb_1.5_Te_3_	Bi_2_Se_0.3_Te_2.7_	Liquid Metal (EGaIn)	Graphene nanoplatelet/EGaIn/PDMS	100	6	5.4		No change in resistance	Ramesh et al.,^18^ 2021
Bi_2_Te_3_	Bi_2_Te_3_	Liquid Metal (EGaIn)	Liquid metal embedded elastomer	1000		87	60	Slight increase in resistance	Zadan et al.,^55^ 2020
Bi_2_Te_3_	Sb_2_Te_3_	Dynamic covalent interactions	Dynamic covalent thermoset polyimine	1000	10	0.516		Slight increase in resistance	Zhu et al.,^50^ 2021
Bi_0.5_Sb_1.5_Te_3_	Bi_2_Te_2.8_Se_0.3_	Dynamic covalent interactions and liquid metals	Dynamic covalent thermoset polyimine	1000	3.5	19	93	No noticeable change in resistance	Ren et al.,^56^ 2021
	Cu-doped Ag_2_Se	Viscoelasticity of the PI substrate	Polyimide	1000	5	8000	50	Resistance change less than 5%	Hou et al.,^60^ 2022
Bi_0.5_Sb_1.5_Te_3_	Bi_2_Te_3_	Liquid Metal (EGaIn)	Ecoflex 00–30	50	11		32.5	No significant change in resistance	Chen et al.,^52^ 2019
Bi_2_Te_3_	Sb_2_Te_3_	Dynamic covalent interactions and liquid metals	Dynamic covalent thermoset polyimine	1000	30	154	10	Resistance change about 1.6%	Zhu et al.,^57^ 2023
PbTe/SWCNTs	Pb_0.97_Bi_0.03_Te/SWCNTs/PEI	Sunlight-driven healable PU	PU	10000			60	Slight increase in resistance	Song et al.,^58^ 2023
CNTP	PEI-CNTP	Dynamic hydrogen bonds in PAA	PAA Hydrogel	100			10	Slight change in resistance	Liu et al.,^59^ 2021
Bi_2_Te_3_	Bi_2_Te_3_	Liquid Metal (EGaIn)	EGaIn/Ecoflex 00-30	2000		115.4	10	No change in resistance	Han et al.,^53^ 2024

By using dynamic covalent thermoset polyimine both as a substrate and as an encapsulation layer, together with liquid metal compounds, TEGs can achieve improved self-healing and recyclable properties, significantly increasing their durability.^50,56^ Zhu et al.^50^ developed a recyclable, self-healing, and stretchable device by fabricating the junctions between commercial thermoelectric Bi_2_Te_3_ and Sb_2_Te_3_ materials with the low-resistance liquid metal EGaIn and using dynamic covalent thermoset polyimine as the encapsulation material. When their device was damaged, bond exchange reactions within the polyimine network created covalent bonds throughout the interface, contributing to the healing of the polymer matrix. The device showed very high performance with its 50% mechanical stretchability and a normalized output power density of 1.08 μW cm^–2^.K^2^, surpassing previous studies on stretchable TEGs.

Ren et al.^56^ have developed a self-healing, reusable, reconfigurable annular device that can be stretched by up to 120% (Figure 4d). Their device’s self-healing capability was achieved through the fluidity of liquid-metal interconnections and bond exchange reactions within the dynamic covalent thermoset polyimine network. When the liquid-metal wiring and polyimine substrate were damaged, the separated interfaces could be reconnected. The liquid-metal interconnects quickly restored electrical conductivity, thanks to its fluidic nature. The bond exchange reactions facilitated the formation of new covalent bonds at the interface, resulting in a fully healed device. When they integrated the device into an LED and subjected it to the cut and heal test, they also showed that the LED turns off when it is cut and becomes operational when it is brought into contact. To increase the thermal conversion efficiency of the device, a wavelength-selective film of metamaterial has also been inserted into the cold part of the device, which helps to ensure that the temperature difference hardly changes even when exposed to sunlight.

Zhu et al.^57^ developed a flexible thermoelectric device with recyclable and self-healing properties by incorporating a boron nitride/polyimine thermally conductive film as the substrate, bulk thermoelectric legs connected by liquid metal electrodes, and polyimine elastomer for encapsulation. Importantly, their device exhibited excellent self-healing and recyclable properties due to dynamic covalent bonds within the stretchable polyimine matrix. Even after healing or recycling, there was no significant degradation in thermoelectric performance. They also created a personal thermal management system with an automatic temperature regulation feature. This system was designed to keep the body temperature within a comfortable range, adapting to changing room temperatures. Additionally, it served as a healthcare function, particularly in situations such as fever or a sprained ankle.

Song et al.^58^ fabricated a TEG with semiconductor nanomaterials and carbon nanotubes composites (PbTe/SWCNT as p-type, Pb_0.97_Bi_0.03_Te/SWCNT/PEI as n-type). The use of sunlight-driven self-healing polyurethane (PU) as a substrate gave their flexible thermoelectric device the ability to self-heal. This innovation not only provided a practical solution for repairing damage during human daily activities but also aligns with environmental sustainability, utilizing sunlight as a clean and readily available energy source. Here, the self-healing mechanism involves sunlight-induced reactions of both disulfide and hydrogen bonds in main chain of PU. The PU substrate plays a role in initiating crack closure through hydrogen bonds, while the subsequent dynamic exchange reaction of disulfide bonds, under sunlight, further contributes to the repair. The strong interaction between the PU substrate and the p-type composite film facilitated the recovery of the thermoelectric performance in the damaged film. The healing efficiency of the scratched p-type composite films was measured at 85.5% when exposed to sunlight. However, under dark conditions, the healing efficiency dropped to 50.9%. The repair was more effective in sunlight than in the dark because, in the dark, only hydrogen bonds are involved in repairing the crack. The self-healing efficiency of the n-type composite film varied under sunlight and dark conditions with an efficiency of 91.0% and 55.8%, respectively. These values, which are slightly higher than those of the p-type film, indicate that both the hydrogen and disulfide bonds in the PU substrate and the hydrogen bonds in the polyethylenimine (PEI) contribute to the overall effectiveness in repairing cracks. After 10,000 cyclic bends, the resistance of both the self-healing p-type and n-type films showed negligible variations. This indicates that both types of self-healing films have excellent cyclic bending stability.

Liu et al.^59^ constructed a TEG using a microwavy architecture that combines intrinsically nonstretchable thermoelectric films with an elastic hydrogel substrate. They used carbon nanotube papers (CNTP) as p-type and PEI-doped CNTP as n-type thermoelectric materials. The hydrogel consists of a covalently cross-linked poly(acrylic acid) (PAA) network with molecular chains that interact with each other through dynamic hydrogen bonds and exhibit both high stretchability and self-healing properties. The designed microwavy architecture extended these properties from the hydrogel substrate to the device, resulting in a highly stretchable TEG with inherent self-healing capacity. The developed TEG can stretch by up to 300% without compromising its thermoelectric performance and can self-heal damage through self-healing of the hydrogel and realignment of the wavy structure.

Hou et al.^60^ developed a TEG by using copper (Cu)-doped silver selenide (Ag_2_Se) thermoelectric films. They coated these films on polyimide (PI) substrate by magnetron sputtering. This TEG exhibited a high power factor of 20.8 μW cm^–1^ K^–2^ at room temperature and achieved a power density of 80 W m^–2^ at a temperature difference of 50 K. The self-healing ability of the Cu-doped Ag_2_Se films is based on the viscoelastic properties of the PI substrate, which allows the closure of microcracks induced by bending. This viscoelastic healing mechanism is particularly advantageous for wearable electronics, as it enables the device to maintain its original properties despite structural damage. The TEG demonstrated superior flexibility, with less than a 5% change in relative resistance after 1,000 bending cycles, and its resistance returned to the initial state within 10 h after bending.

While advances in TEGs have focused on various components, the substrate materials remain a critical element in improving the overall performance of the device. Recent innovations have introduced dynamic covalent polyimines, graphene nanoplatelets and PDMS-based substrates that offer high thermal conductivity, stretchability and self-healing capability. Future developments could target substrates that offer even more effective thermal management and flexibility, such as biocompatible or multifunctional materials. These advances could facilitate the seamless integration of TEGs into wearables, medical devices and smart textiles, enabling autonomous power generation and real-time health monitoring. Substrate development will continue to play an important role in improving the efficiency, durability and scalability of TEGs.

### 5.3 Self-Healable and Stretchable Thermoelectric Materials

**Figure 5:**
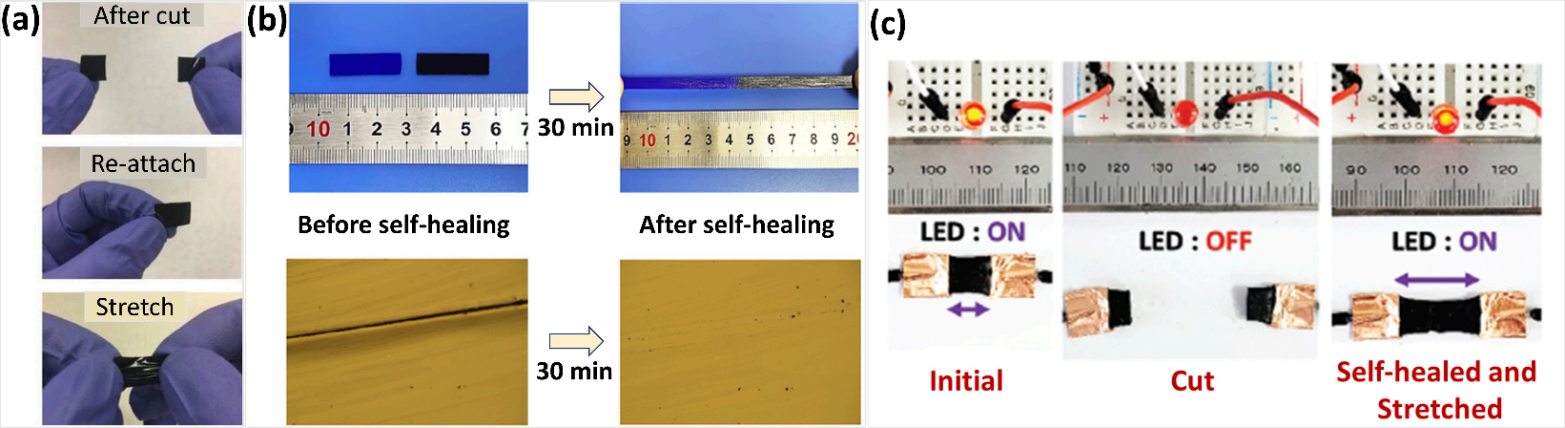
(a) Composite film after cutting, reattachment, and stretching developed by Kee et al.^28^ Reprinted from ref (28). Copyright 2019 The Authors. (b) Self-healing behavior of PI −30 developed by Xu et al.^64^ Adapted with permission from ref (64). Copyright 2021 American Chemical Society. (c) Organic–inorganic ionic TE composites with self-healing properties developed by Malik et al.^62^ Adapted with permission from ref (62). Copyright 2021 John Wiley and Sons.

In addition to self-healing interconnects and substrates, intrinsically self-healing stretchable thermoelectric materials have also been investigated for the continuous operation of wearable electronic devices. In a 2019 study, a self-healing and stretchable thermoelectric device was developed using 3D printing techniques, incorporating Triton X-100 as a healing agent, dimethyl sulfoxide (DMSO) as an additive, and a ternary poly(3,4-ethylenedioxythiophene) doped with polystyrenesulfonate (PEDOT:PSS) composite^28^ (Figure 5a). When a LED was connected to the film and the performance of the device was tested, it was observed that the LED did not switch off immediately after cutting, nor did it switch off afterward. This proves the fast self-healing ability (about 1s) of the developed composite. Observations indicate that the self-healing behavior varies depending on the thickness of the film and the width of the damaged area. The device fabricated with this self-healing composite in this study exhibits a stretchability of up to 35% while retaining more than 85% of its performance, allowing it to function even after repeated cuts. It has also been demonstrated that the film did not exhibit any self-healing behavior and its performance was not maintained unless a surfactant, Triton X-100 was used.

In another study,^61^ a stretchable and self-healing p-type thermoelectric composite material, SIS(styrene-isoprene-styrene)/P3BT(poly(3-butylthiophene))/BCF(bis(cyclopentadienyl)cobalt(II)) was developed. The self-healing mechanism was induced by heat and pressure. The incorporation of 19 mol % BCF resulted in a composite exhibiting a stretchability of 400% under a stress of 14 MPa. It was demonstrated that cutting, healing, and stretching did not significantly reduce the thermoelectric performance. In the same study, the self-healing behavior of the film was observed by physically damaging it in two different ways: shallow scratches and deep scratches. The film with deep scratches showed a thermoelectric performance close to its undamaged state after heat treatment and the application of finger pressure for self-repair. On the other hand, it was shown that the film with a shallow scratch was able to heal itself completely. In another study from 2020, self-healing, flexible and stretchable ternary ionic thermoelectric materials made of polyaniline, anionic polyelectrolyte and phytic acid (PANI:PAAMPSA: PA) were developed for the first time.^9^ The materials presented in this study performed quite well compared to other studies in terms of stretchability up to 750% and thermoelectric performance during repeated self-healing cycles. The ionic thermoelectric performance of the ternary ionic materials remained consistent even when subjected to 50% strain and multiple cutting-healing processes (30 cycles), marking the initial showcase of such remarkable performance. It has been demonstrated that the produced film can autonomously self-heal without any external stimuli. It has also been noted that as scratches expand, self-healing time also increases. Malik et al.,^62^ on the other hand, developed a self-healing stretchable material by adding inorganic SiO_2_ nanoparticles to the ternary composite inorganic–organic ionic polyaniline: poly(2-acrylamido-2-methyl-1-propanesulfonic acid): phytic acid (PANI:PAAMPSA: PA). It has been demonstrated that the produced film can autonomously self-heal without any external stimuli after 25 cutting-healing cycles. The reversible interactions facilitating self-healing and stretching properties were attributed to the surface hydroxyl groups on SiO_2_ nanoparticles within the PANI:PAAMPSA: PA matrix. No degradation in performance was observed with this material up to a strain of 100% (Figure 5c). Akbar et al.^63^ proposed a ternary self-healing and stretchable composite material consisting of 1-ethyl-3-methylimidazolium trifluoromethanesulfonate (EMIM:OTf), poly(vinylidene fluoride-*co*-hexafluoropropylene)(PVDF-HFP), and fluorosurfactant (FS). The proposed composite showed very high thermoelectric performance, even under 75% strain and after multiple shear conditioning processes.

Extensive research has also been carried out on ion- gels to develop thermoelectric materials that are both self-healing and stretchable. Xu et al.^64^ have prepared self-healing and stretchable polyurethane ionogels (Figure 5b). The ionogels they prepared contain an ionic liquid called EMIM:DCA (1-ethyl-3-methylimidazolium:dicyanamide) and polyurethane. With this material, there was no significant loss of thermoelectric performance even at 50% elongation. The ionogel’s ability to rapidly self-heal from external damage is attributed to the dynamic nature of its boronic ester bonds, ionic bonds, and hydrogen bonds. These bonds can undergo continuous breaking and reforming under ambient conditions, facilitating the efficient repair of the material.

Liu et al.^65^ have developed ionic thermoelectric ionogels with self-healing capabilities by incorporating a highly amorphous polymer that produces reversible ion-dipole interactions with ionic salts, enabling self-healing properties. The ion- gels, which have stretchable and self-healing properties for ionic thermoelectric applications, consist of a polymer matrix, PVDF-HFP (poly(vinylidene fluoride-*co*-hexafluoropropylene)) with low crystallinity, an ionic liquid, EMIMTFSI (1-ethyl-3-methylimidazolium bis(trifluoromethylsulfonyl)imide), and certain salts that serve as thermal power regulators. The mechanism that gives these ionogels self-healing properties is based on the reversible ion-dipole interactions between the liquid electrolyte and the PVDF-HFP polymer, which has a dipole moment. These interactions act like dynamic bonds, enabling the self-healing process. This research is the first case in which self-healing ionic thermoelectric materials with negative thermopower (n-type) have been developed. The obtained thermoelectric ionogels have been demonstrated to rapidly regain their mechanical and thermoelectric properties through cut-healing tests.

Fu et al.^66^ synthesized ionic hydrogels labeled as PAA–PEO-NaCl (Poly(acrylamide))-poly(ethylene oxide)-sodium chloride), featuring various salt concentrations, using a single-step photoinduced polymerization method and ion balancing. The ionic hydrogels exhibited remarkable mechanical resilience, adhesion, and self-healing properties due to reversible hydrogen bonding interactions and chain entanglement. Under ambient conditions, the electrochemical and thermoelectric performance of these hydrogels swiftly recovered from physical damage. They have also stated that higher humidity accelerates the movement of polymer chains and facilitates the reformation of hydrogen bonds, leading to faster healing of the ionic gel. The mechanical healing process of damaged device was fully restored within a 24-h time frame. This study has demonstrated that multifunctional ionic hydrogels can be used for heat-to-electricity conversion in wearable thermoelectric applications. Table 2 shows recent studies on self-healing materials used in wearable TEGs.

**Table 2:** Self-Healing Thermoelectric Materials

**Thermoelectric Material**	**Figure of Merit (ZT)**	P**ower Factor** (μW m^–1^ K^–2^) After Healing	**Maximum Stretchability**	**Self-Healing Cycle**	**Healing Time**	**Performance After Healing**	**Healing Mechanism**	**Ref**
PEDOT:PSS/DMSO/Triton X-100		2.1	Up to 35%	10	1 s	Retaining >85% of its initial power factor	Hydrogen bonding-based self-healing	Kee et al.,^28^ 2019
SIS/P3BT/BCF	0.00126	∼0.2	∼400%		30 min	No significant change in thermoelectric(TE) performance (Retain its original TE performance for multiple shallower scratched)	Heat and Pressure-induced self-healing	Jeong et al.,^61^ 2020
PANI:PAAMPSA:PA	1.04	1600	750%	30	<4 min for 25 mm-wide scratch	Retain its original TE performance	Dynamic hydrogen bonds and electrostatic interactions	Akbar et al.,^9^ 2020
SiO_2_ NPs/PANI:PAAMPSA:PA	∼3.74 (for 80% relative humidity)	∼5.99	720%	25	2 min	No significant change in TE performance	Reversible dynamic hydrogen bonds and electrostatic interactions	Malik et al.,^62^ 2022
(PVDF-HFP/EMIM:OTf)	2.34 (for 90% relative humidity)	1570	505%	15	30 min	No noticeable performance degradation	Dynamic ion–dipole interactions and heat-induced self-healing	Akbar et al.,^63^ 2022
EMIM:DCA/PU	0.99 ± 0.3		300%	1	30 min	No noticeable performance degradation	Dynamic boronic ester bonds, ionic bonds, and hydrogen bonds	Xu et al.,^64^ 2021
EMIMTFSI/PVDF-HFP/LiBF_4_			∼1700%	3	24 h	No noticeable performance degradation	Reversible ion-dipole interactions	Liu et al.,^65^ 2022
PAA/PEO/NaCl	∼0.2	∼0.2	∼1200%		24 h (mechanical healing)	No noticeable performance degradation	Reversible dynamic hydrogen bonds	Fu et al.,^66^ 2023
PAAc/XG/Bi_2_Se_0.3_Te_2.7_			256%	5	2 s	Retaining >99% of its initial power output	Hydrogen bonding-based self-healing	Li et al.,^67^ 2022
PEDOT:PSS/PVA/PEG		5.6 ± 0.8	119%	4		Retaining 84% of its power factor	Reversible boron ester bonds, Coulombic forces, and hydrogen bonds	Liao et al.,^68^ 2022
GaSn/B–Sb_2_Se_3_		2.932		5		Retaining 96% of its power factor	Liquid metals (GaSn) and heat-induced self-healing	Kim et al.,^69^ 2023
PEO/LiTFSI-EmimBF_4_			300%	2	15 min	Retaining more than 97% of the ionic seebeck coefficients	Dynamic and reversible physical cross-linking between lithium ions and ether oxygen of PEO chains	Zhao et al.,^70^ 2023
PEO/LiTFSI/EmimCl			2000%	3	30 min (for 60% and 90% humidity)	Retaining its ionic seebeck coefficient	Dynamic Li–O coordination and hydrogen bonding	Zhao et al.,^71^ 2024

Li et al.^67^ synthesized a stretchable and self-healable hybrid thermoelectric material consisting of PAAc (Poly(acrylic acid)), XG(Xanthan gum) and Bi_2_Se_0.3_Te_2.7_ by in situ polymerization method. The material they developed could autonomously self-heal within 2 s without the need for any external stimuli. Furthermore, they have indicated that the self-healing process accelerates when an external force is applied to the damaged material. After 1000 bending cycles with a radius of 6 mm, 98.1% of the electrical conductivity is retained, which indicates the flexibility of the device. Their device also maintained 99% of its power output after cutting.

Liao et al.^68^ developed an intrinsically self-healing, wearable, and all-organic thermoelectric composite. This composite consists of poly(3,4-ethylenedioxythiophene)/polystyrenesulfonate (PEDOT/PSS)/poly(vinyl alcohol) (PVA) hydrogel and polyethylene glycol (PEG). The self-healing capability is facilitated by reversible hydrogen bonding interactions within the PVA and borax cross-linked network, enabling the material to recover its structure and conductivity after physical damage. This allows the composite to re-establish electrical pathways, which is critical for thermoelectric performance recovery. After self-healing, their composite retains about 78% of its original electrical conductivity and 84% of its power factor, indicating only minor performance degradation.

Kim et al.^69^ created self-healing thermoelectric composites by utilizing liquid metals derived from Ga–Sn and Sb_2_Se_3_ thermoelectric materials. The composite was endowed with self-healing capability through the use of liquid metal which also increases electrical conductivity. The power factor of the composite remained above 96% after five cutting-healing cycles. The fabricated composite was heated to approximately 130 °C after being cut, initiating the self-healing process, given that the melting point of Ga–Sn alloys is 166 °C.

Zhao et al.^70^ synthesized a poly(ethylene oxide) (PEO)-based n-type ionogel. This ionogel consists of a PEO polymer matrix, a lithium salt (lithium bis(trifluoromethanesulfonyl)imide, LiTFSI), and an ionic liquid (Emim tetrafluoroborate, EmimBF_4_. This ionogel comprises a PEO polymer matrix, a lithium salt (lithium bis(trifluoromethanesulfonyl)imide, LiTFSI), and an ionic liquid (Emim tetrafluoroborate, EmimBF_4_). Its autonomous self-healing capability arises from the dynamic and reversible physical cross-linking between lithium ions and the ether oxygen in the PEO chains. Cuts on this ionogel heal rapidly upon contact, disappearing almost completely within 15 min. Additionally, the ionogel is recyclable. The recycling process involves dispersion in ethanol, where the polymer chains recoordinate due to noncovalent interactions as the solvent evaporates, restoring their original structure and properties.

Zhao et al.^71^ also developed a unique ionogel composed of poly(ethylene oxide) (PEO), lithium bis(trifluoromethanesulfonyl)imide (LiTFSI), and 1-ethyl-3-methylimidazolium chloride (EmimCl), to achieve phase separation and exceptional mechanical properties. This ionogel exhibits autonomous self-healing enabled by dynamic lithium-ether and hydrogen bonding interactions. With a high stretchability of up to 2000%, it also maintains consistent conductivity across multiple cut-healing cycles. Surface scratches of up to tens of micrometers on this ionogel fully heal at room temperature. The ionogel’s healing is accelerated in high-humidity conditions, achieving full repair within 30 min at 60% and 90% humidity, promising for flexible, durable thermoelectric applications.

Recent studies demonstrate significant progress in developing self-healing, stretchable thermoelectric materials, showing remarkable durability and performance retention. For instance, materials were able to maintain over 98% conductivity after extensive bending cycles, while others retained more than 96% of their power output following multiple cutting and healing processes. Fast self-healing properties were observed, even under ambient conditions, thanks to dynamic bonding mechanisms. Stretchability of up to 1700% was achieved without compromising thermoelectric efficiency. These advancements highlight the potential for creating robust, long-lasting wearable devices that can autonomously heal, making them ideal for energy-harvesting applications in future technologies.

## 6 Conclusions

The self-healing property ensures long-term reliability by preventing device failure after damage and opens the door to advanced technologies. As self-healing materials become increasingly important, their applications are expanding to triboelectric nanogenerators, sensors, energy storage and electronics. In wearable thermoelectric systems, they improve efficiency and enable continuous operation under load. This is particularly critical in healthcare, where an uninterruptible power supply is essential for real-time health monitoring in wireless wearables.

The rise of liquid metals in soft electronics, particularly in wearable thermoelectric devices, has revolutionized flexibility and performance. Bismuth-based materials, while offering strong thermoelectric properties, are less suitable for human use due to their stiffness and toxicity. Recent advances in self-healing devices using liquid metals encapsulated in elastomers have improved safety, but the risk of breakage remains. The future lies in the development of lightweight, flexible and nontoxic organic thermoelectric materials that can self-repair. Biocompatibility, especially in healthcare wearables, is critical for safe, long-term use without the risk of toxicity or irritation. With the development of advanced materials, wearable TEGs that can continuously power wireless microelectronics will revolutionize wearable technology. This innovation opens up new possibilities for self-powered devices, leading to a future of smart, adaptable and seamlessly integrated wearables that ensure the future of energy is both sustainable and wearable.

Future research in the field of self-healing materials should focus on improving biocompatibility and long-term safety, especially for implantable applications. Optimizing the composition of hydrogels and EGaIn-based systems to improve performance, durability and reduce toxicity is crucial. Research into novel additives, cross-linking methods and scalable production techniques will address industry needs, such as cost-effectiveness and mass production, while ensuring environmental compatibility, washability and breathability — key factors for wearable and biomedical devices. Rigorous testing to ensure nontoxic interactions with human skin will further support their adoption in medical applications. Addressing the challenges of functionality, scalability and sustainability will ultimately drive progress in wearable technologies.
